# Cardiopulmonary exercise testing as a predictor of complications in oesophagogastric cancer surgery

**DOI:** 10.1308/003588413X13511609954897

**Published:** 2013-03

**Authors:** LH Moyes, CJ McCaffer, RC Carter, GM Fullarton, CK Mackay, MJ Forshaw

**Affiliations:** NHS Greater Glasgow and Clyde, UK

**Keywords:** Cardiopulmonary exercise testing, Postoperative complications, Oesophagogastric cancer, Anaerobic threshold

## Abstract

**Introduction:**

An anaerobic threshold (AT) of <11ml/min/kg can identify patients at high risk of cardiopulmonary complications after major surgery. The aim of this study was to assess the value of cardiopulmonary exercise testing (CPET) in predicting cardiopulmonary complications in high risk patients undergoing oesophagogastric cancer resection.

**Methods:**

Between March 2008 and October 2010, 108 patients (83 men, 25 women) with a median age of 66 years (range: 38–84 years) underwent CPET before potentially curative resections for oesophagogastric cancers. Measured CPET variables included AT and maximum oxygen uptake at peak exercise (VO_2 _peak). Outcome measures were length of high dependency unit stay, length of hospital stay, unplanned intensive care unit (ICU) admission, and postoperative morbidity and mortality.

**Results:**

The mean AT and VO_2_ peak were 10.8ml/min/kg (standard deviation [SD]: 2.8ml/min/kg, range: 4.6–19.3ml/min/kg) and 15.2ml/min/kg (SD: 5.3ml/min/kg, range: 5.4–33.3ml/min/kg) respectively; 57 patients (55%) had an AT of <11ml/min/ kg and 26 (12%) had an AT of <9ml/min/kg. Postoperative complications occurred in 57 patients (29 cardiopulmonary [28%] and 28 non-cardiopulmonary [27%]). Four patients (4%) died in hospital and 21 (20%) required an unplanned ICU admission. Cardiopulmonary complications occurred in 42% of patients with an AT of <9ml/min/kg compared with 29% of patients with an AT of ≥9ml/min/kg but <11ml/min/kg and 20% of patients with an AT of ≥11ml/min/kg (*p*=0.04). There was a trend that those with an AT of <11ml/min/kg and a low VO_2_ peak had a higher rate of unplanned ICU admission.

**Conclusions:**

This study has shown a correlation between AT and the development of cardiopulmonary complications although the discriminatory ability was low.

Surgical resection remains the best chance of cure for patients presenting with oesophagogastric cancer.^1^ There is morbidity associated with oesophagectomy and gastrectomy, with cardiopulmonary complications responsible for a substantial proportion of postoperative morbidity and mortality.^2^ The 2010 National Oesophago-gastric Cancer Audit reported that at least one complication occurs in 30% of patients after an oesophagectomy and in 19% after a gastrectomy.^3^ Complications have a negative impact on survival and quality of life after surgery, and new perioperative strategies should aim to optimise technique and minimise post-operative complications.^4,5^

Surgery places severe stresses on a patient’s cardiopulmonary reserve, increasing oxygen demand by approximately 40%.^6^ The Confidential Enquiry into Perioperative Deaths concluded that most perioperative deaths in elderly patients arose from pre-existing cardiorespiratory disease.^7^ Most patients undergoing pre-operative assessment for major surgery such as an oesophagectomy or a gastrectomy will have echocardiography, spirometry or a dobutamine stress test to assess cardiopulmonary performance. However, it is clear that while these screening tests may identify some high-risk patients, they do not provide accurate objective information or guide management to reduce postoperative morbidity and mortality.

Over the past few years, there has been interest in the role of cardiopulmonary exercise testing (CPET).^7^ CPET is a simple, non-invasive, cost-effective test that can be performed in either an inpatient or outpatient setting, providing the clinician with an integrated assessment of a patient’s cardiovascular and pulmonary system in less than one hour.^9^ CPET measures oxygen uptake at increasing levels of work and can measure cardiopulmonary performance objectively at rest and under stress, determining the patient’s physiological capacity to cope with the demands of surgery.

Older *et al* have shown that all postoperative cardiopulmonary deaths occur in patients with an anaerobic threshold (AT) of <11ml/min/kg and/or with significant myocardial ischaemia on CPET.^6^ CPET results have been used to stratify patients undergoing major surgery, to guide preoperative optimisation, to predict postoperative cardiac complications after abdominal surgery and, in some centres, to assess whether borderline patients should undergo lung resection.^8,10^ The aim of this study was to assess the value of CPET in predicting cardiopulmonary complications in high-risk patients undergoing oesophagogastric cancer resection.

## Methods

### Patients

Between March 2008 and October 2010, 180 consecutive patients underwent CPET as a formal fitness assessment prior to consideration for resection after discussion at the multidisciplinary cancer meeting. In our preoperative staging algorithm, CPET was done before positron emission tomography – computed tomography in patients with oesophageal cancer and before laparoscopy in those with gastric cancer. Patients considered for radical surgical treatment had clinical evaluation, chest x-ray, electrocardiography, pulmonary function tests and echocardiography. Stress echocardiography and thallium myocardial perfusion imaging were performed only in selected patients after review by a cardiologist. CPET was performed along with existing measures of fitness and was not the sole arbiter of fitness.

### Cardiopulmonary exercise testing

CPET was performed in the respiratory function laboratory in the Glasgow Royal Infirmary with a doctor and full resuscitation equipment present. The ZAN^®^ 600 (nSpire Health, Hertford, UK) and the Ergoselect bicycle ergometer (Ergoline, Bitz, Germany) were used for the tests. During the test, patients were exposed to incremental physical exercise on a bicycle ergometer to their maximally tolerated level, which was determined by either exhaustion or, more often, the development of symptoms (pain or breathlessness). Several variables were recorded throughout the procedure including blood pressure, cardiac activity via electrocardiography, and inspiratory and expiratory gases (to calculate oxygen uptake and carbon dioxide output). Two key measurements can be determined from these data, VO_2_ peak (oxygen uptake at peak exercise) and AT, indicating the point at which anaerobic metabolism is inadequate to maintain high energy phosphate production in exercising muscles, forcing anaerobic metabolism to make up the deficit.^11^

### Surgery

Patients received the standard care under the responsible consultant surgeon with administration of prophylactic antibiotics and thromboembolic prophylaxis. The operation performed depended on tumour site, tumour size and surgeon preference. Patients were nursed in a high dependency unit (HDU) postoperatively. A diatrizoic acid swallow was performed on day 7 after the oesophagectomy and total gastrectomy to assess anastomotic healing.

### Outcome measures

Length of HDU stay, length of hospital stay, unplanned intensive care unit (ICU) admission, and postoperative morbidity and mortality were recorded. Postoperative morbidity was divided into cardiopulmonary and non-cardiopulmonary complications. Cardiopulmonary complications were defined according to *Common Terminology Criteria for Adverse Events*.^12^ Non-cardiopulmonary complications included septic (wound infection, intra-abdominal collection), anastomotic or operative complications. AT measurements were divided into three categories: <9ml/min/kg, ≥9ml/ min/kg but <11ml/min/kgn and ≥11ml/min/kg.

### Statistical analysis

Variables were grouped using standard thresholds. Categorical data were compared using chi-squared tests. Continuous data were compared using Student’s t-test and the Mann–Whitney U test as appropriate. The relationship between length of hospital stay and CPET measurements was assessed by linear regression. A *p*-value of <0.05 was deemed statistically significant. Analysis was performed using SPSS^®^ version 18.0 (SPSS, Chicago, IL, US).

## Results

**Figure 1:**
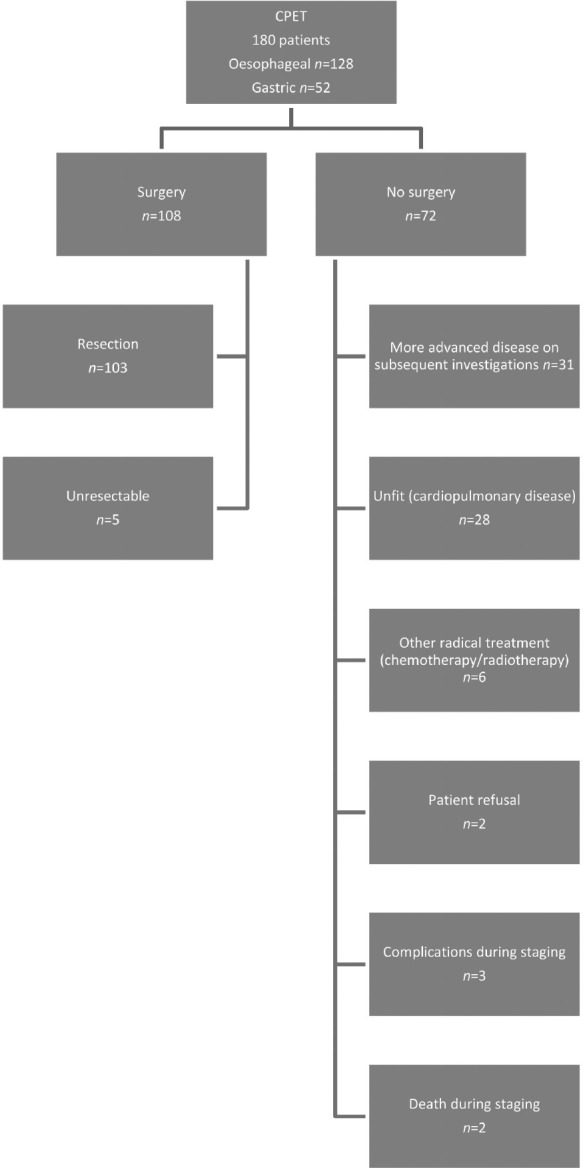
Flowchart of all patients undergoing cardiopulmonary exercise testing (CPET)

Of the 180 patients, 108 (83 men, 25 women) with a mean age of 66 years (standard deviation [SD]: 9 years, range: 38–84 years) were suitable for surgical resection while 72 were not suitable for the reasons shown in Figure 1. The baseline demographics of the patients undergoing resection are shown in Table 1.

**Table 1:** Baseline demographics of patients undergoing resection

		Patients
Sex	Male	83 (77%)
	Female	25 (23%)
Age	<65 years	46 (43%)
	65–74 years	49 (45%)
	≥75 years	13 (12%)
Deprivation category	1–2 (deprived areas)	15 (14%)
	3–4	47 (43%)
	5–7 (affluent areas)	46 (43%)
Site of cancer	Oesophageal	65 (60%)
	Gastric	43 (40%)
Thoracotomy	Yes	40 (37%)
	No	68 (63%)
Operation	Transhiatal oesophagectomy	24 (22%)
	Transthoracic oesophagectomy	40 (37%)
	Total gastrectomy	15 (14%)
	Subtotal gastrectomy	24 (22%)
	Inoperable at laparotomy	5 (5%)
Neoadjuvant chemotherapy	Yes	78 (72%)
	No	30 (28%)
Pathology	Adenocarcinoma	96 (89%)
	Squamous carcinoma	8 (7%)
	Leiomyoma	2 (2%)
	Gastrointestinal stromal tumour	1 (1%)
	High grade dysplasia	1 (1%)

The mean VO_2_ peak in the 103 patients undergoing oesophagogastric resection was 15.2ml/min/kg (SD: 5.3ml/ min/kg, range: 5.4–33.3ml/min/kg). The mean AT was 10.8ml/min/kg, with 57 patients (55%) having an AT of <11ml/min/kg and 26 patients (25%) an AT of <9ml/min/ kg. The mean AT in patients with significant cardiorespiratory co-morbidity and therefore deemed unfit for surgery after outpatient clinical assessment was 8.6ml/min/kg (SD: 1.9ml/min/kg). This was significantly lower than those patients undergoing resection (*p*<0.001). There was no significant difference in the mean AT or VO_2_ peak according to the operative approach, type of operation, tumour location or presence of thoracotomy.

Postoperative complications occurred in 57 patients (55%) with 29 (28%) developing cardiopulmonary complications and 28 (27%) developing non-cardiopulmonary complications (Table 2). The mean HDU stay and total length of hospital stay was 9.0 days (SD: 5.9 days, range: 0–53 days) and 20.2 days (SD: 15.4 days, range: 2–97 days) respectively. Twenty-one patients (20%) required an unplanned ICU admission and the in-hospital mortality rate was 3.9% (4 deaths). Three of these patients died from cardiopulmonary complications (mean AT 9.9ml/min/kg); two patients developed pneumonia and subsequent respiratory failure requiring ventilation (AT 13.7ml/min/kg and 7.7ml/min/kg respectively), and the third patient developed cardiac failure and died in the ICU (AT 8.3ml/min/kg). The fourth death was due to sepsis after an anastomotic leak (AT 9.8ml/min/kg).

**Table 2:** Postoperative complications in patients undergoing oesophagogastric resection

	Transthoracic oesophagectomy (*n*=24)	Transhiatal oesophagectomy (*n*=40)	Total gastrectomy (*n*=15)	Subtotal gastrectomy (*n*=24)
All complications*	**20**	**36**	**6**	**9**
Cardiopulmonary**	**14**	**22**	**3**	**2**
Arrhythmia	1	2	0	0
Cardiac ischaemia/infarction	1	2	0	1
Cardiac failure	1	0	1	0
Atelectasis	1	0	0	0
Pneumonia	5	10	2	1
Pleural effusion	1	1	0	0
Pulmonary embolism	0	1	0	0
Respiratory failure requiring ICU	4	6	0	0
**Non-cardiopulmonary**	**6**	**14**	**3**	**7**
Anastomotic leak	2	8	3	1
Sepsis	1	2	1	5
Operative complication	3	4	0	1

ICU = intensive care unit

* Some patients had more than one complication

** As defined by *Common Terminology Criteria for Adverse Events* ^12^

**Figure 2:**
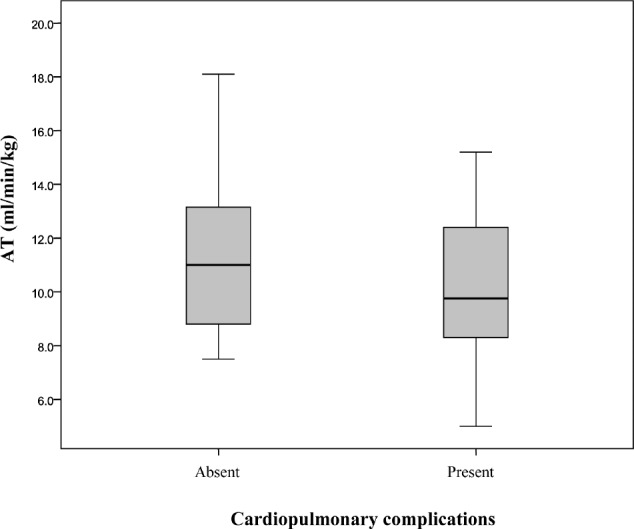
Mean anaerobic threshold (AT) and the presence of complications

**Figure 3:**
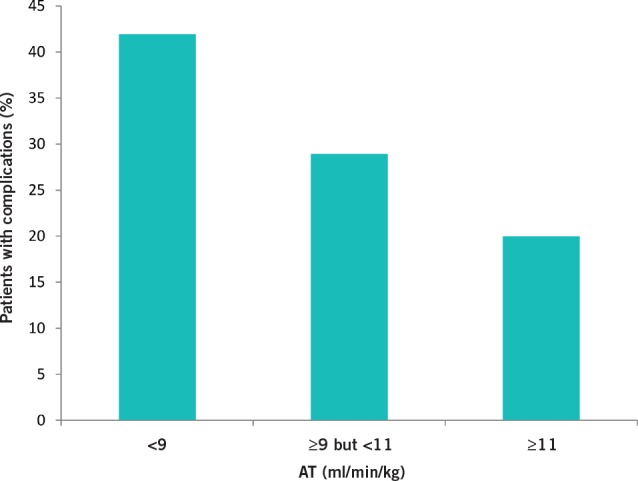
Cardiopulmonary complications in each anaerobic threshold (AT) group

The mean AT in those with cardiopulmonary complications was 9.9ml/min/kg compared with a mean of 11.2ml/ min/kg in patients with no cardiopulmonary complications (*p*=0.05) (Fig 2). The majority of cardiopulmonary complications occurred in those with a low AT (*p*=0.04) (Fig 3). A low VO_2_ peak was not associated with an increased risk of cardiopulmonary complications (14.6ml/min/kg vs 16.6ml/ min/kg, *p*=0.07). No significant increase was found in unplanned ICU admission rates or length of hospital stay in patients with an AT of <11ml/min/kg or a low VO_2_ peak.

Table 3 summarises the relationships between the presence of cardiopulmonary complications and the studied variables. Complications were associated with oesophageal tumours, particularly in patients requiring thoracotomy, unplanned ICU admissions and longer inpatient hospital stay. A low AT and VO_2_ peak seemed to identify patients at risk.

**Table 3:** The relationship between the studied variables and cardiopulmonary complications in patients undergoing resection

		No CP complications (*n*=74)	CP complications (*n*=29)	*p*-value
Age (years)	<65 65–74 ≥75	33 (45%) 30 (40%) 11 (15%)	11 (38%) 16 (55%) 2 (7%)	0.93
Sex	Male Female	55 (74%) 19 (26%)	24 (83%) 5 (17%)	0.26
Site of tumour	Gastric Oesophageal	35 (47%) 39 (53%)	4 (14%) 25 (86%)	**0.001**
Thoracotomy	Yes No	24 (32%) 50 (68%)	16 (55%) 13 (45%)	**0.02**
Intensive care unit admission	Yes No	8 (11%) 66 (89%)	13 (45%) 16 (55%)	**<0.001**
Reoperation	Yes No	7 (10%) 67 (90%)	3 (10%) 26 (90%)	0.574
Hospital length of stay (days)	≤14 15–28 >28	40 (54%) 25 (34%) 9 (12%)	8 (28%) 12 (41%) 9 (31%)	**0.006**
In-hospital mortality	Yes No	1 (1%) 73 (99%)	3 (10%) 26 (90%)	0.06
Anaerobic threshold (ml/min/kg)	<9 ≥9 but <11 ≥11	15 (20%) 22 (30%) 37 (50%)	11 (38%) 9 (31%) 9 (31%)	**0.04**
Mean VO_2_ peak (ml/min/kg)		16.6 (SD: 5.4)	14.6 (SD: 5.1)	0.08

CR = cardiopulmonary; SD = standard deviation

**Figure 4:**
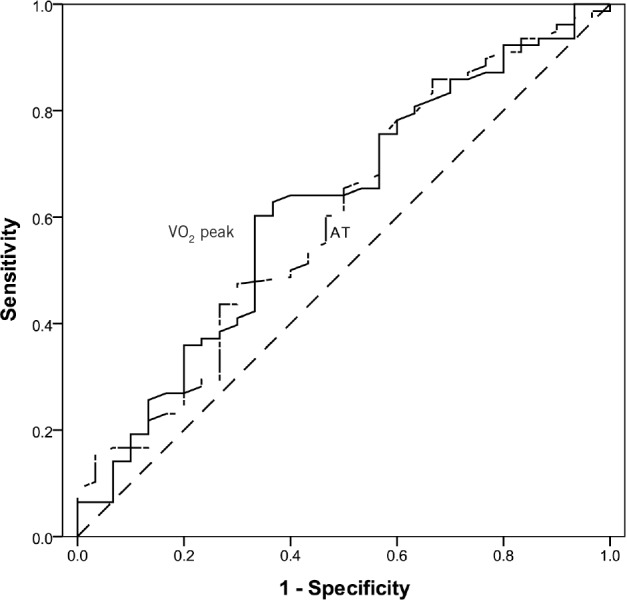
Receiver operating characteristic curve for predicting cardiopulmonary complications from anaerobic threshold (AT) and maximum oxygen uptake at peak exercise (VO_2_ peak). The diagonal reference line indicates no discrimination.

Figure 4 shows the receiver operating characteristic (ROC) curves for VO_2_ peak and AT as predictors of cardiopulmonary complications. The area under the ROC curve for VO_2_ peak and AT was 0.62 (95% confidence interval [CI]: 0.50–0.74, *p*=0.06) and 0.60 (95% CI: 0.48–0.72, *p*=0.08) respectively. Using Older *et al*’s recommended AT cut-off value of 11ml/min/kg,^6^ the sensitivity and specificity of AT in predicting postoperative cardiopulmonary complications in our cohort was only 45% and 30% respectively (*p*=0.09). We therefore examined a variety of alternative cut-off values, and found that an AT of 9ml/min/kg had a sensitivity of 74% and a specificity of 57% (*p*=0.04), and this was able to predict the development of postoperative cardiopulmonary complications in our population.

## Discussion

This study investigated the relationship between preoperative CPET outcome measures and the development of postoperative complications in 103 patients undergoing resection for oesophagogastric cancer. The complication and mortality rates in our patients were similar to those published for the rest of the UK.^3^

The variables derived from CPET were AT and VO_2_ peak. Other studies report a mean AT of 13.9ml/min/kg^13^ but we found a lower mean AT in our population (10.8ml/min/kg). Older’s original study assessed CPET variables in an elderly population with mixed pathologies (mainly colorectal cancer and vascular surgery) in Australia and defined a cut-off AT value of 11ml/min/kg as a useful discriminator for cardiorespiratory reserve.^6^ However, 55% of our patients had an AT of <11ml/min/kg. In order to study more severe impairment present in our population, we examined three categories of AT: <9ml/min/kg, ≥9ml/min/kg but <11ml/min/kg and ≥11ml/min/kg.^14,15^ Using this classification system, we have shown that patients with a low AT carry a higher risk of developing postoperative cardiopulmonary complications. ROC curve analysis suggested that a reducing AT is a progressive marker of increasing risk. In our population, an AT cut-off of 9ml/min/kg seems to be a more appropriate value.

A study from 2012 analysing the effects of a low cardiopulmonary reserve and outcome, particularly anastomotic leak, after a pancreaticoduodenectomy suggested an AT of 10.1ml/ml/kg was an appropriate cut-off level in their cohort using ROC analysis.^16^ Our study has confirmed that different populations may require individual AT levels rather than the global cut-off of 11ml/min/kg as described by Older *et al*.^6^ However, what seems to be clear is that patients with a low cardiorespiratory reserve are at increased risk of postoperative morbidity.

Older *et al* assessed AT initially in relation to major surgery in elderly patients, suggesting a risk grading and treatment protocol.^6^ They showed that an AT of <11ml/min/kg was associated with higher morbidity and mortality rates. To date, our study is the first that suggests an association between a low AT and the development of post-operative complications following oesophagogastric cancer surgery.

In our unit, CPET results are not used primarily to determine which patients may have an operation although they can provide objective evidence to support clinical concern. Studies have shown that preoperative optimisation with beta-blockers and statins can improve the cardiorespiratory function in high risk surgical patients.^17,18^ Perioperative ‘goal directed therapies’ can also help to reduce postoperative morbidity and mortality. Perioperative CPET may highlight those patients with a low AT, allowing preoperative optimisation with a cardiology review and drug therapy if appropriate, and retesting after initiation of the treatment. The information from CPET may play a part in the consent process, informing patients and their families that they are at a higher risk of postoperative cardiopulmonary complications. A preoperative AT may allow surgeons and anaesthetists to determine whether patients with a low AT should be managed in the ICU rather than the HDU postoperatively.

Our study has some limitations. The study population is heterogeneous with patients undergoing oesophagectomy and gastrectomy, and some requiring thoracotomy. Our study numbers are small, which may account for the lack of statistical significance associated with unplanned ICU admission, and it is difficult to determine primary and secondary complications, for example atrial fibrillation as a secondary response to an anastomotic leak.

## Conclusions

CPET provides an objective, overall assessment of cardiorespiratory reserve in patients undergoing major surgery such as oesophagogastric cancer resection. We have shown that patients with a low AT have a higher risk of developing cardiopulmonary complications. Further work is required in larger cohorts to corroborate these findings and formulate appropriate management plans for high-risk patients.
